# Improving Optical Measurements: Non-Linearity Compensation of Compact Charge-Coupled Device (CCD) Spectrometers

**DOI:** 10.3390/s19122833

**Published:** 2019-06-25

**Authors:** Münevver Nehir, Carsten Frank, Steffen Aßmann, Eric P. Achterberg

**Affiliations:** 1 GEOMAR Helmholtz Centre for Ocean Research Kiel, Wischhofstr. 1-3, 24148 Kiel, Germany; mnehir@geomar.de; 2 HAW Hamburg, Faculty of Life Sciences, Ulmenliet 20, 21033 Hamburg, Germany; Carsten.Frank@haw-hamburg.de; 3 Helmholtz Centre Geesthacht, Institute of Coastal Research, Max-Planck-Str. 1, 21502 Geesthacht, Germany; Steffen.Assmann@km.kongsberg.com; 4 Kongsberg Maritime Contros GmbH, Wischhofstr. 1, 24148 Kiel, Germany

**Keywords:** charge-coupled device, compact spectrometer, optical measurements, spectrometer errors, non-linearity correction

## Abstract

Charge-coupled device (CCD) spectrometers are widely used as detectors in analytical laboratory instruments and as sensors for in situ optical measurements. However, as the applications become more complex, the physical and electronic limits of the CCD spectrometers may restrict their applicability. The errors due to dark currents, temperature variations, and blooming can be readily corrected. However, a correction for uncertainty of integration time and wavelength calibration is typically lacking in most devices, and detector non-linearity may distort the signal by up to 5% for some measurements. Here, we propose a simple correction method to compensate for non-linearity errors in optical measurements where compact CCD spectrometers are used. The results indicate that the error due to the non-linearity of a spectrometer can be reduced from several hundred counts to about 40 counts if the proposed correction function is applied.

## 1. Introduction

**Figure 1:**
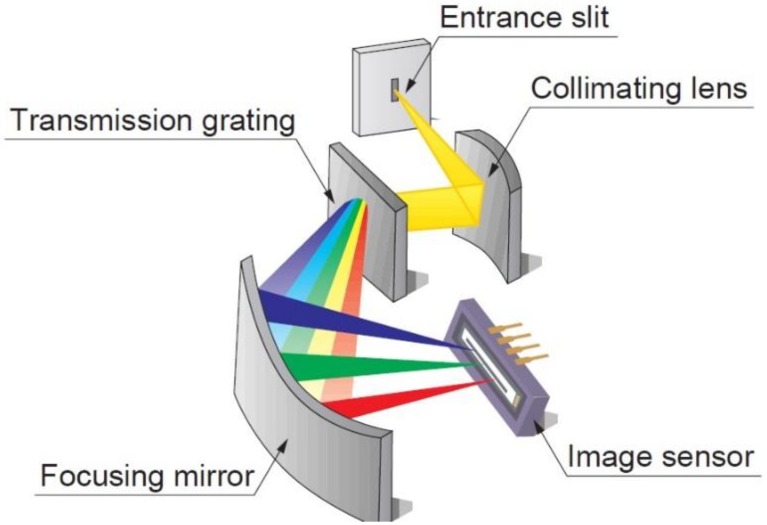
Schematic of a Hamamatsu C10082CA miniaturized spectrometer [1].

Charge-coupled device (CCD) spectrometers are compact detectors that provide spectral information about the light that reaches the image sensor component. The devices usually consist of an entrance slit, a collimating lens, a transmission grating, a focusing mirror, a CCD line/image sensor, and some electronics. A schematic view of a CCD spectrometer is shown in Figure 1. While there are various configurations available where a reflective rather than a transmissive grating is applied, or the entrance slit is omitted and instead an aperture of a fiber bundle is used, the error sources remain the same.

When using CCD spectrometers, error sources at each pixel that cause a deviation of the corresponding signal from the correct value have to be considered. The CCD spectrometers usually have a dark current, which has to be determined and subtracted from each spectrum before the data is used. For older CCD spectrometers, one has to account for blooming of super-saturated pixels [2]. These errors can be readily compensated for, or are negligible for the more recent CCD sensors. Li et al. [3] studied the effect of temperature on the response of CCD spectrometers, and obtained a deviation of less than 1% between the measured and calculated responses at randomly selected temperatures between 5 and 40 °C. Furthermore, the non-linear behavior of the CCD line has to be considered as a significant measurement error source. Experiments have indicated that the non-linearity at an intensity of about 50,000 (of a maximum of 65,535) counts is in the range of up to 1000 counts on the intensity scale, or up to 0.04 absorption units on the absorbance scale, using a Hamamatsu C10082CA mini-spectrometer [4]. Non-linearities in the light intensity response of CCD spectrometers greater than 10% were found in recent studies and the corresponding corrections resulted a non-linearity of less than 0.5% [5,6]; in one study a custom-designed spectrometer [5] was used and in the other an Avantes Avaspec-ULS2048L spectrometer and a StellarNet Blue-Wave spectrometer [6] were used. To the best of our knowledge, Ocean Optics Inc. is the only supplier that provides non-linearity correction software (OOINLCorrect, Ocean Optics Inc., Dunedin, FA, USA) for their CCD spectrometers, which provides linearities >99.8% [7]. 

Other miniature spectrometers are equipped with a photodiode detector array (PDA) instead of a CCD detector and offer some advantages. There are different semiconductor technologies used to build PDAs, one is the complementary metal-oxide-semiconductor field-effect transistors (CMOS) technology. These PDAs are generally more linear compared to CCD detectors and have a wider dynamic range. However, they are less sensitive and therefore less common [8] and were not tested in this work.

Charge-coupled device-based spectrometers are used in a variety of applications, including spectrophotometric flow injection analysis using only one or two wavelengths for simple absorption measurements [9], multiple wavelengths for multi-component determination [10], and for the direct determination of a certain analyte in the context of a complex background signal [11]. Specific fields of these applications are the determination of dissolved phosphate [9], dissolved trace metals [10], pH [4,12,13], and hyper-spectral determination of nitrate, bromide, and colored dissolved organic matter [11,14] in seawater.

Another important application field of CCD spectrometers is in space instrumentation, where they are used to explore astronomical objects [15]. The CCD spectrometers are also used in meteorology, for example, to detect changes in spectral UV irradiance due to changes in the thickness of the ozone layer [16]. While these applications are very different, the challenges with the spectrometers are similar [17].

Further uses of CCD are in imaging applications combined with a single photon camera and a multichannel detector, which is straightforward and significantly improves the performance of time-correlated photon counting [18].

Other optical sensors that use a CCD-detector but rely on a different optical setup may also be affected by the non-linear response of the CCD line. One example is a method that allows time resolved measurements based on a stroboscopic light source and a revolving grating [19].

Compared with the conventional bench-top spectrophotometers, CCD spectrometers have the advantages of being low-cost, small in size, light in weight, robust, and suitable for high-speed data assimilation (milli-seconds) with a steady performance [20,21].

In this study, we describe and evaluate a non-linearity correction method for a CCD spectrometer that only depends on the intensity of the signal and is independent of integration time and wavelength. We aim to broaden the applicability of the CCD spectrometers. Our findings are illustrated for each pixel using a Hamamatsu C10082CA mini-spectrometer.

## 2. Theory

### 2.1. Design of a CCD Spectrometer

**Figure 2:**
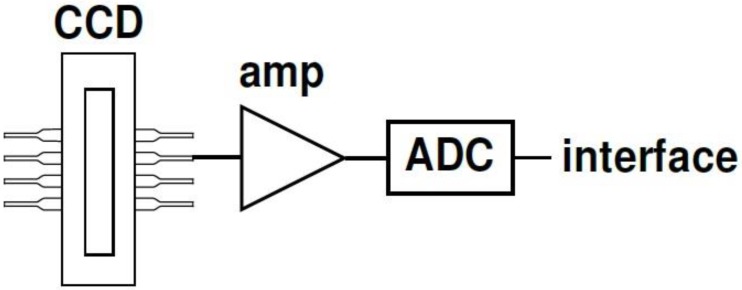
Simplified diagram of the basic internal circuit that connects the charge-coupled device (CCD) line with the interface (RS232 or USB) of the computer. The CCD line is connected via an amplifier (amp) with the analog-to-digital converter (ADC). The latter is either part of a microcontroller or connected to a microcontroller that communicates with the computer.

In this study, a Hamamatsu S10420-1106-01 CCD line was connected via an amplifier to a 16-bit analog-to-digital converter (ADC). The ADC was connected to a microcontroller that controlled the measurement parameters (e.g., integration time) and transmitted data to the interface via USB and/or RS232. Figure 2 shows a simple schema, which is nevertheless a good approximation of most of the possible circuit variations. 

### 2.2. Signal Composition

There are several error sources for CCD spectrometers, which can also be found in common monochromator-based desktop instruments. We describe the sources below.

#### 2.2.1. ADC Offset

**Figure 3:**
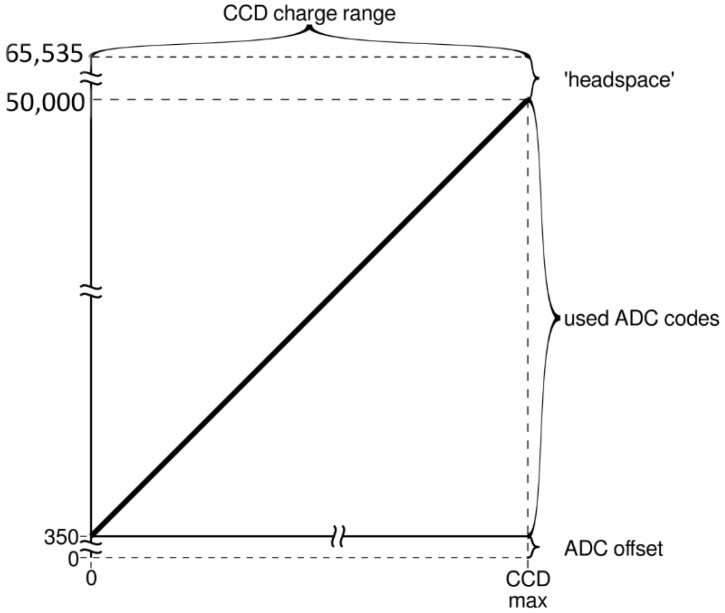
Diagram showing how all possible CCD output values (after amplification; X-axis) are mapped onto the value range of the analog-to-digital converter (ADC; Y-axis). As the zero value of the CCD (and the amplifier) may vary with time, temperature, and/or other factors, this zero value is mapped to a value that lies well inside the value range of the ADC.

An ADC device is used to convert an analog to a digital signal, and its resolution is determined by the number of bits (e.g., a 16-bit device has 2^16^ discrete digital output values). An ADC offset (*w***_ADC_**) is used to shift the lowest possible signal into the range of voltages that can be converted by the ADC (Figure 3). It can be expected that a similar limitation exists at the upper end of the sensitivity range, called ‘headspace’ in Figure 3. This, however, is inconsequential for the presented approach as the working range is defined up to 50,000 counts.

#### 2.2.2. Dark Current

The dark current is typically dominated by thermal generation of random holes and electrons in the photon sensitive layer of the CCD line. Space radiation and other charge carriers are among the other sources [22,23]. Independent of the cause, the dark current should be the same for all pixels. In this study, the signal received from the spectrometer during ‘dark’ is described as follows:

(1) $$I_{dark^{*}}\left(\lambda \right)=\omega_{ADC}+\mathrm{NL}\left(I_{dark}\left(\lambda \right)\right)\cdot t\;$$

where *w***_ADC_** is the ADC offset, *I***_dark_**(λ) is the current that is generated by the random generation of holes and electrons, *t* is the integration time, and NL is the non-linearity of the detector. However, the term ’dark current’ is commonly used for the actual intensities that are transmitted by the spectrometer during ’dark’ measurements. This value is referred to as *I*_dark*_(λ) in Equation (1).

#### 2.2.3. Non-Linearity

Non-linearity describes the difference between the changes in the detector signal in comparison to the changes in the light intensity. The signal of an ideal detector would proportionally increase with an increasing light intensity. The slope between light intensity and detector counts would thus be constant. Any deviation from this behavior is called non-linearity. The error caused by non-linearity is a systematic error that can seriously diminish the quality of measurement results in some specific cases, such as high accuracy measurements or relative measurements between two or more wavelengths [4].

Any existing non-linearity is typically the result of the combined non-linearities of the CCD pixel, amplifier, and ADC offset [24]. Since all CCD pixels of one chip are expected to have very similar properties, it can be assumed that one non-linearity correction function is valid for all pixels.

#### 2.2.4. Blooming

Blooming is the effect where electrons are leaking from one CCD pixel to neighboring pixels. This charge transfer only occurs if the blooming pixel is saturated. Blooming can be prevented with the aid of an anti-blooming function of the CCD spectrometer, which, however, affects the linearity of the pixels as well as their sensitivity [25].

The Hamamatsu sensor used in this study (C10082CA, CCD line: S10420-1106-01) has an anti-blooming function.

#### 2.2.5. Stray Light

Stray light, *I**_stray_***(λ), is unintended light within the detector that may reduce the signal to noise ratio. The stray light may have the following sources: (i) scattered light from internal CCD walls and input optics of the spectrometer, (ii) scattered light from the dispersive element, usually a diffraction grating, (iii) inter-reflections, and (iv) light coupling [26]. Salim et al. [26] and Zong et al. [27] suggested the use of a spectrally tunable laser or a lamp with a high spectral emission in the UV region combined with a monochromator to gain data that can then be used to correct for stray light. Both light sources can be used to generate a narrow-band monochromatic light, which is then used as a light source for a spectrometer. The stray light is determined as the intensities measured at neighboring as well as all wavelengths, after taking the spectral resolution of the slit and grating into consideration. The impact of the error depends on the intensity of the light source, the extent of absorbance, and on the specific spectrometer used. Preliminary estimates based on experiments involving spectrophotometric pH measurements in seawater as described in Aßmann et al. [4] indicated a stray light error of approximately −0.0004 pH units. More detailed information can be found in the studies mentioned above [4,26,27].

#### 2.2.6. Uncertainty of the Integration Time

The linearity of the integration time compared to the value that is termed integration time by the software is a critical factor in the method proposed in this work. Inside the spectrometer, the integration time is managed by the microcontroller that uses an internal timer to close the shutter. This timing is therefore only dependent on the quality of the firmware implementation. The expected effects would be a constant offset that accounts for the processing time of the interrupt handling (in the order of <10^−6^ s) and a drift in the clocking frequency of the oscillator used to drive this timer. The first cannot be distinguished from the ADC offset and is therefore part of this value. The second should only play a significant role if the spectrometer is exposed to a strong temperature drift, which can be ruled out in our study.

#### 2.2.7. Wavelength Calibration

It is required to conduct a wavelength calibration when using a CCD spectrometer to determine the corresponding pixel location of each wavelength prior to use [28]. We used a high-end desktop spectrometer (Perkin Elmer Lambda 950) as a secondary standard to determine the wavelength accuracy of our CCD spectrometer. The accuracy of the wavelength calibration of the compact CCD spectrometer was better than any deviation we could determine (<±0.2 nm).

#### 2.2.8. Overall Signal Composition

In summary, the raw signal (*I***_raw_**(λ) in counts) has the following function:

(2) $$I_{raw}\left(\lambda \right)=\omega_{ADC}+\mathrm{NL}\left(I_{dark}\left(\lambda \right)+I_{stray}\left(\lambda \right)+\;I\left(\lambda \right)\right)\cdot t$$

where *I*(λ) is the current caused by the generation of holes and electrons by photons. It is produced by the light-sensitive area of a highly doped silicon (Si) semiconductor. If a photon hits an electron in the light-sensitive area, the electron is lifted into the conductive band and instantly drawn to the cathode by the internal electrical field, which is generated by the doped Si areas. The electrons are accumulated in a charge well and measured after amplification with the ADC. The investigation of stray light is beyond the scope of this study. In our applications, *w***_ADC_** is subtracted from each signal. This leads to the following equation:

(3) $$I_{raw}\left(\lambda \right)=\mathrm{NL}\left(I_{dark}\left(\lambda \right)+\;I\left(\lambda \right)\right)\cdot t$$

## 3. Materials and Methods

### 3.1. Experimental Setup

**Figure 4:**
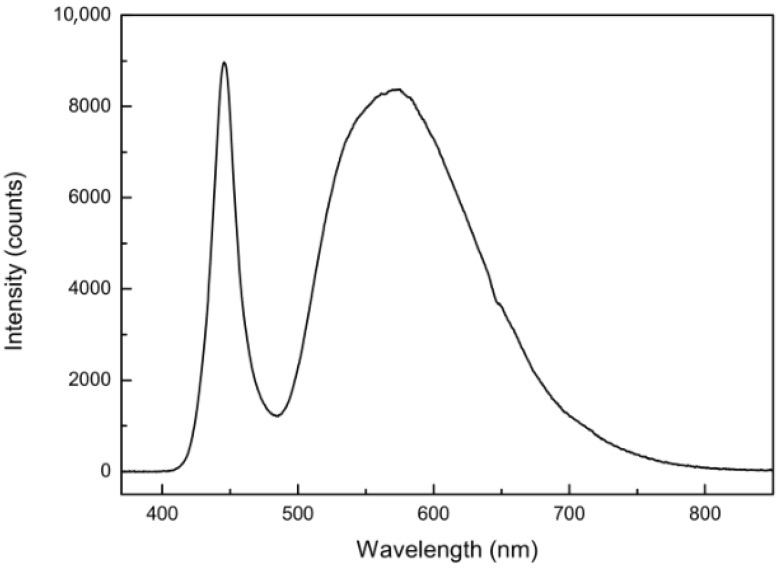
Light intensity spectrum of the white LED used in this study.

Quantification of the measurement errors of the mini-spectrometer used in this study was performed with the following setup. A white Futurlec Star-LED (spectrum shown in Figure 4) combined with a simple, custom-made precision current source were used as a light source to produce broadband spectra; the spectral range covered 364–893 nm. A Hamamatsu C10082CA spectrometer with a Hamamatsu S10420-1106-01 CCD line were used to record the intensity counts at each pixel (Table 1). A combination of light fibers was used to reduce the light intensity of the LED to a degree where the minimal integration time of 10 ms yielded a maximum light intensity of about 1200 counts (445 nm) in comparison to a wavelength where the LED had no intensity (380 nm).

**Figure 5:**
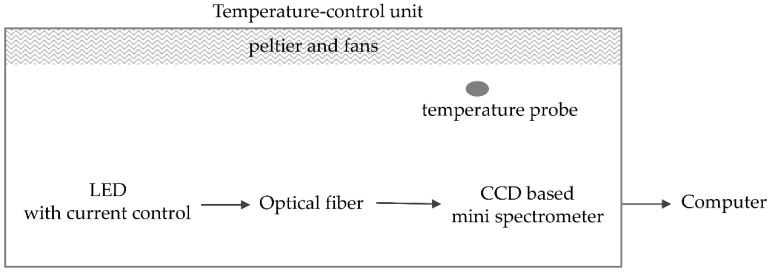
Experimental setup with instrumentation in the cool box for the quantification of the non-linearity of the miniature spectrometer.

The LED with its current source, the optical fiber, and the spectrometer were mounted in a modified 48 L cool box (Mobicool W48). The lid of the cool box was removed and replaced with a homemade device with more cooling power and space for cables (Figure 5). Care was taken to isolate the experiment inside the box from ambient light. The experiment was conducted at different temperatures (20 °C, 25 °C, and 30 °C ± 0.1 °C).

All the measurements were controlled using routines written in the Python programming language, version 3.7.1, [30] on a Linux operating system using custom-made device drivers. The specific device drivers ensured that the data from the spectrometer was not modified by the manufacturer’s device driver. All data analysis was undertaken using the R (2018) language for statistical computing [31].

### 3.2. Independent Linearity Test

To assess the non-linearity of the spectrometer a simple experiment can be performed. The results of this experiment are independent of the integration time. It is assumed that an increase in the integration time leads to a proportional increase in the signal at each pixel. A model for this behavior of uncorrected intensities *I***_i_** obtained at different integration times (*t***_i_**) is described in Equation (4), which leads via rearrangement (Equation (5)) to the ratio *f* (Equation (6)):

(4) $$\frac{I_{1}\left(\lambda \right)}{t_{1}}=\;\frac{I_{2}{\left(\lambda \right)}_{\;}}{t_{2}}$$

(5) $$\frac{I_{1}{\left(\lambda \right)}_{\;}}{I_{2}{\left(\lambda \right)}_{\;}}=\;\frac{t_{1}}{t_{2}}$$

(6) $$f=\;\frac{I_{1}\left(\lambda \right)}{I_{2}{\left(\lambda \right)}_{\;}}$$

**Figure 6:**
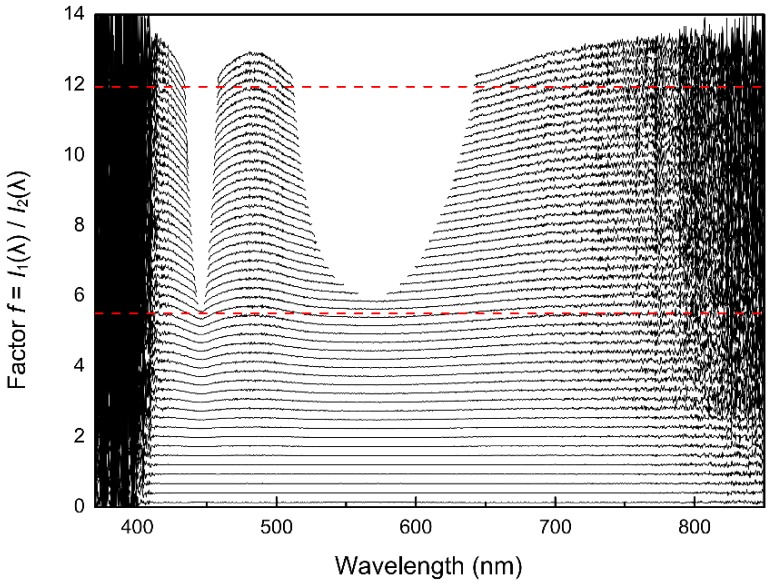
Qualitative illustration of the non-linearities of the CCD detector. The ratios *f* of *I***_1_**(λ) to *I***_i_**(λ) are plotted versus the wavelengths (nm) for several integration times. In theory, the f ratios should be constant for all wavelengths of a spectrum. The dashed red lines are the expected characteristics for two arbitrarily chosen integration times, the black lines are the observed characteristics for several integration times (compare also with Figure 11).

Figure 6 shows *f* ratios plotted against wavelength. The *f* ratios are calculated from spectra taken from the same light source (see Figure 4) with different integration times. Since the only difference between each single spectrum is the change in the integration time, the intensities should scale accordingly, resulting in a constant value of *f* for all pixels.

The quality of the result of the determination of *f* strongly depends on the accuracy of the determination of the ADC offset. Any deviation in that value may result in inconclusive results as Equations (4)–(6) rely on a linear relationship (through zero) between the intensities and integration times. A second approach could be to subtract the intensities of the so called ‘dark pixels’ (pixels that do not receive any light) from all pixels prior to their use in this linearity test. This approach was also tested and yielded comparable results, however, it should only be used for the linearity tests as the intensities of the dark pixels varied with intensity, which led to a reduced accuracy during the linearity calibration.

**Figure 7:**
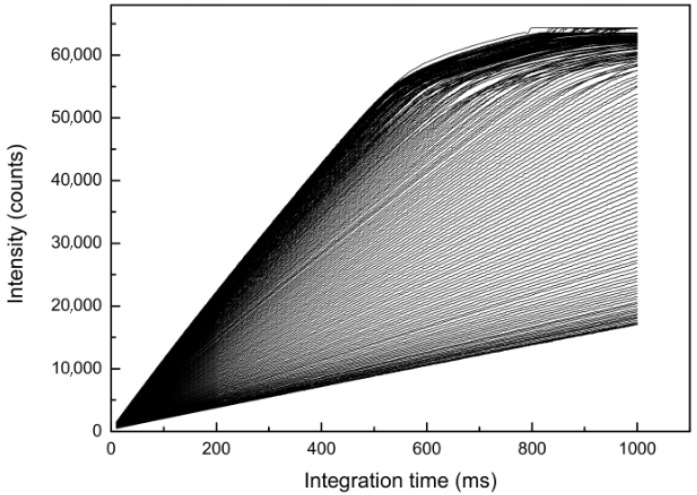
Pixel intensities (counts) versus integration time (ms). A single line represents data from one pixel. Data from 200 representative pixels (equally spaced between 400–800 nm) are shown. All data lines appear to be linear up to 50,000 counts and then deviate strongly from the ideal line.

All the points above 50,000 counts were omitted due to strong increases of the non-linearity above 50,000 counts (Figure 7). Despite the qualitative nature of this way to display the data, it becomes evident that a significant non-linearity exists.

## 4. Results and Discussion

### 4.1. ADC Offset

The ADC offset is the signal which represents the number of counts that are read at a theoretical integration time of zero (Figure 3). This value reflects the signal of the CCD line and a signal that was more or less randomly selected by the circuit designer (see Figure 3).

The ADC offset value is important for the determination of the non-linearity correction function, as the ADC offset has to be subtracted from all values prior to further operations. The subtraction of the ADC offset allows the forcing of the linear regression through zero at a zero integration time. Forcing the linear regression through zero using dark measurements is superior as it prevents inaccuracies caused by thermal noise associated with a regression using low light intensities.

**Figure 8:**
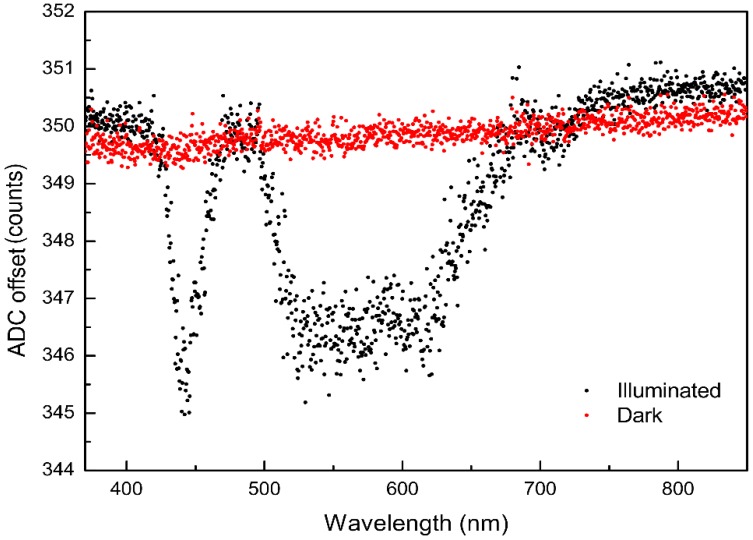
ADC offset in counts for all pixels of the CCD line. The black dots were for an enabled light source (illuminated) and the red dots were determined for a disabled light source (dark).

The estimation of the ADC offset relies on the assumption that the non-linearity between the signal of the CCD line and ADC is not too large when only a small part of the dynamic range of the CCD is used. To obtain the ADC offset, the LED was switched off and spectra were collected at different integration times leading to intensities ranging between 350 and 1200 counts. These spectra were then used to determine the intensity (in counts) at zero integration time using a linear regression for each pixel (red dots in Figure 8). Alternatively, the ADC offset can be obtained by determining the lowest possible signal, when the LED is switched on, at wavelengths without light intensity (e.g., 380 nm, black dots in Figure 8). Both ways should theoretically yield the same result. While we found a difference of one count and a smaller drift over the whole range of pixels, this deviation lies well within the theoretical precision of the ADC (±0.5 counts) and thus can be neglected. In this study, the ADC offset amounted to 350 counts at 20 °C.

### 4.2. Correction of the Non-Linearity

The non-linearity correction is based on the assumption that the integration time is highly reproducible and determined by the time that was actually selected. First, the data were collected by measuring spectra at about 1800 different integration times. The variation in integration times ranged from 10 to 1000 ms, with at least 30% of the illuminated pixels being saturated at 1000 ms. Blooming was prevented by the anti-blooming function of the CCD detector. At least 25 spectra were obtained using each integration time and the pixel-specific data were averaged.

Figure 7 shows a selection of pixels for which intensity values were plotted against integration time. As a linear relationship between the intensity and the integration time did not exist for intensity values above 50,000 counts, this threshold was defined as an upper limit of the usable data range. Of the remaining data, the intensities of pixels obtained at the longest integration times were averaged for the intensity range between 47,000 and 50,000 counts in order to construct a referential raw intensity (*I***_raw_**) versus integration time (*t*) curve.

A linearization was undertaken using two points, with the first point being the origin of coordinates and the second point being more or less freely chosen. A linear regression was used to determine the slope.

The resulting regression function was used to calculate a corrected intensity (*I***_corr_**) for each integration time, independent of wavelength. *I***_corr_** represents the expected result (a linear relationship between intensity and integration time) while *I***_raw_** represents the measured values a_0_ to a_8,_ which represent values of regression coefficients; both datasets (one pair of *I***_corr_** and *I***_raw_** for all integration times) can be used to find a suitable correction function. The models tested for the correction functions were simple polynomial functions up to the ninth degree:

(7) $$I_{corr}=a_{0}\cdot I_{raw}+a_{1}\cdot \;I_{raw}^{2}+a_{2}\cdot \;I_{raw}^{3}+\ldots +a_{8}\cdot I_{raw}^{9}$$

**Figure 9:**
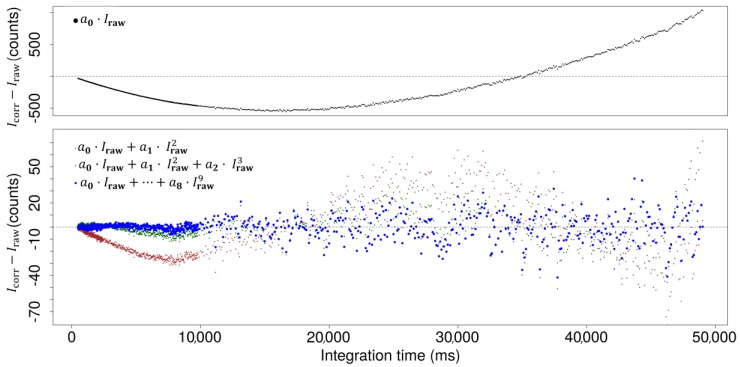
Differences between corrected and referential intensities for selected polynomials (*I***_corr_** − *I***_raw_**; Y-axis) versus integration time (ms). The panel above shows a wider range of intensity counts for the first-degree polynomial function, while the panel below shows a narrower range for the second-, third-, and ninth-degree functions.

The linear model function (‘lm’) in the R programming language [31] was used to fit the data to the model. The differences between the corrected data and the theoretical optimum are given in Figure 9.

It is evident that with an increasing degree of the polynomial function, the estimation error decreases. However, degrees above nine did not yield significantly better results, and even the difference between the third and the ninth degrees was quite small (Figure 9).

**Figure 10:**
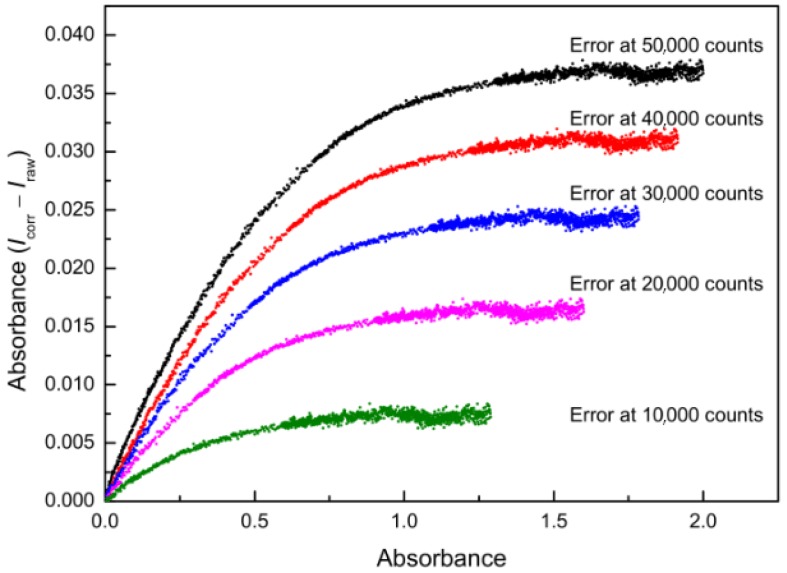
Differences between corrected and referential raw intensities in terms of absorbance error values (Y-axis) at different absorbance levels (X-axis).

The optimum is a horizontal line, as the ratio *I***_1_**(λ)/*I***_2_**(λ) should be constant for all pixels of each spectrum since only the integration time is changed (Figure 6). Differences between corrected (*I***_corr_**) and theoretical optimum (*I***_raw_**) intensities showed a systematic deviation, for example, the absorbance error at an intensity of 50,000 counts amounts to 0.04 absorbance units (Figure 10). After the correction, the deviation of the corrected intensities from the ideal linear line amounts to a maximum of 40 counts at higher intensities. These 40 counts are well below the noise of the detector (200 counts), and do not show a systematic pattern (Figure 9).

### 4.3. Uncertainty in the Integration Time

**Figure 11:**
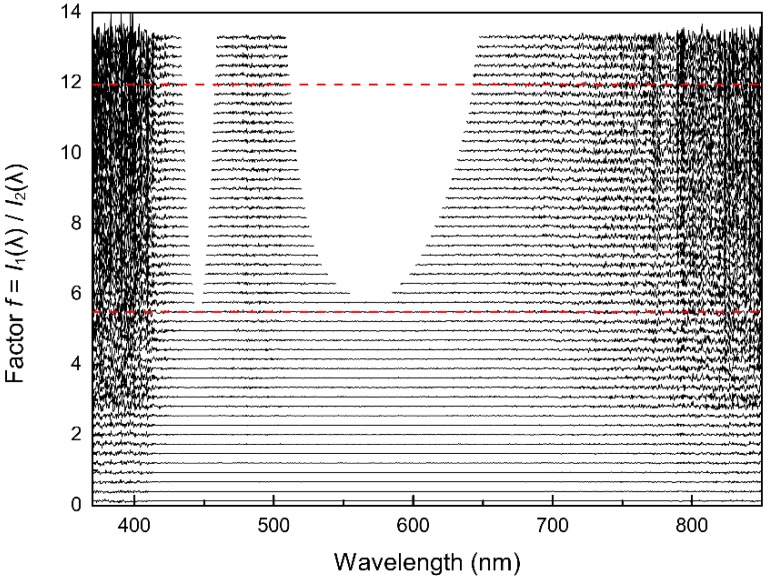
Illustration of the linear dependence of the corrected intensities on the integration time. The ratios *f* of *I***_1_**(λ) to *I***_i_**(λ) are plotted versus the wavelengths (nm) for several integration times. The correction is valid for different intensities at different integration times and wavelengths and thus verifies the applicability of the correction function. The dashed red lines are expected characteristics for two arbitrarily chosen integration times, the black lines are the corrected observed characteristics for several integration times (compare also with Figure 6).

The correction function was applied to raw data collected during the experiment mentioned in Section 4.2. The results in Figure 11 show a significant improvement compared to the data prior to the non-linearity correction (see Figure 6).

The overall improvement compared to the initial state also shows that the initial assumptions about the linearity of the integration time were correct. Any deviation in the reproducibility and scalability of the integration time would produce artifacts in Figure 11, as data of different integration times and intensity ranges are compared with each other in this plot.

### 4.4. Detector Noise

**Figure 12:**
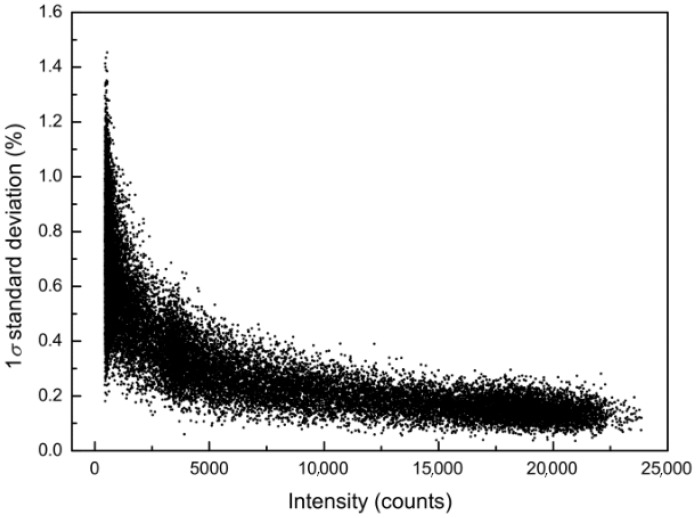
Relative detector noise as a function of intensity (counts) of the CCD detector given as 1 σ standard deviation (%).

Figure 12 shows example data of the relative standard deviation of repeated intensity measurements (N = 10) as a function of intensity. On a relative basis, the error at intensities of around 500 counts is about 30 counts higher (more than 1%) than for intensities of around 20,000 counts, where the error of the intensity lies at around 200 counts (less than 0.25%).

Therefore, the noise-free resolution, which refers to the number of stable ADC bits at a constant input signal, is around 2^11^ counts at low intensities and 2^8^ at medium intensities. While the resolution can be improved by averaging the signal, it would be necessary to average 2^5^ spectra at low intensities and 2^8^ spectra at medium intensities to obtain a true 16-bit resolution.

The full dataset (not shown here) indicates that this behavior is independent of the integration time and only depends on the intensity.

### 4.5. Using Curve Fitting to Reduce the Noise of the Detector

The detector noise can also be reduced by using standard curve fitting procedures (e.g., R’s loess function or polynomials). This curve fitting uses the neighboring pixels (usually 10 pixels or more) of the wavelength of interest to reduce the noise of the target pixel. This procedure also allows for the calculation of wavelengths between two contiguous pixels. However, this method can only be applied under certain conditions (no spikes in the spectrum of the lamp) and only gains a two- or three-fold noise reduction. 

### 4.6. Temperature Dependency

The spectrometer used in this study (Hamamatsu C10082CA) has a small temperature dependency (in this study in the range of 2 counts per °C), but the resulting offset is usually constant as it is independent of the integration time and wavelength. With our spectrometer, the temperature affected the ADC offset, which changed from 350 counts at 20 °C to 338 counts at 25 °C and to 326 counts at 30 °C. The non-linearity correction function remained constant.

### 4.7. Methodological Summary

The method to compensate for the non-linearity effects of CCD spectrometers can be performed using a very simple setup in a temperature-controlled environment. Minimum requirements are a stable temperature (±1 °C) and a stable light source (e.g., a white LED with a highly stable constant current source). Furthermore, the integration times programmed into the spectrometer should be linear and stable with time (see Section 2.2.6). 

The following dataset has to be acquired: Light (LED on) and dark (LED off) spectra at a wide range of integration times; these depend on the capabilities of the spectrometer. In this case, we used 1800 integration times between 10 ms (lowest) and 1000 ms and took at least 25 spectra at each integration time for averaging. One of the integration times was selected and repeated at regular intervals during the measurement to ensure the stability of the system.

Suggested procedure according to Figure 3:

- Determination of the ADC offset: calculate a linear regression of the dark spectra vs. integration time and calculate the dark current (in counts) at an integration time of zero. The result is the ADC offset (see Equation (1)) for the used spectrometer.
- Plot the intensity of a range of pixels versus the integration time to determine the level at which the signal response of the spectrometer becomes obviously non-linear (see Figure 7) and exclude all data above that threshold (e.g., 50,000 counts in the case of the Hamamatsu C10082CA) during all consecutive steps.
- Select a number of the pixels at high integration times where the intensities are close to the threshold and average these pixels to form the reference graph (*I***_raw_**(t)). Subtract *w***_ADC_** from all intensities of this intensity data curve and perform a linear regression (forced through zero). Calculate a corrected intensity (*I***_corr_**) for all integration times of the reference graph.
- There is now a value-pair of *I***_raw_** and *I***_corr_** for all integration times that can be used to formulate a relationship. In the R programming language, Equation (7)
  $(I_{corr}=a_{0}\cdot I_{raw}+a_{1}\cdot \;I_{raw}^{2}+a_{2}\cdot \;I_{raw}^{3}+\ldots +a_{8}\cdot I_{raw}^{9}$) would look like:
  “lm(yNew ~ 0 + y + I(y^2) + I(y^3) + I(y^4) + I(y^5) + I(y^6) + I(y^7) + I(y^8) + I(y^9))” with y = I_raw_ and yNew = I_corr_ (see Section 4.2).
- Use this function to calculate a linear corrected intensity (*I***_corr_**) for each raw intensity (*I***_raw_**) independent of the integration time.

The method described here can be applied to all CCD-based spectrometers that fulfill the requirements described above. The experimental setup as well as the mathematics behind the method are simple. While the data acquisition and processing requires some time and processing power, the resulting correction function computes efficiently. Restrictions apply to applications where the spectrometer is subject to changing temperatures, which can be resolved using a temperature-correction function for the ADC offset.

To the best of our knowledge, only Ocean Optics provides a non-linearity compensation algorithm for its CCD units. The non-linearity correction feature of this software is based on the polynomial regression between the amount of light the detector receives and integration time. When comparing our approach with that provided by the proprietary OOINLCorrect Software [7], there are similarities concerning the key approach of using a change in integration time and subsequent data linearization. However, our approach is universally applicable to all CCD units, openly accessible, and has the following additional differences:

- We determined the ADC offset first, which seems to be a key way of achieving better accuracy at low light conditions. This is done by forcing the linear regression through zero at zero integration time, using dark measurements, which is superior as it prevents inaccuracies caused by thermal noise.
- We excluded data where we knew that the response of the instrument was highly nonlinear (>50,000 counts) (see Figure 7). While this may not be an issue with the Ocean Optics spectrometers, this certainly is an issue for the CCD-based Hamamatsu units. It is possible to use the full range of minimum and maximum counts of the spectrometer. However, it should be considered during data analysis that the error behavior is different for different ranges of counts.
- We provided criteria as to which pixels to select for averaging to give a better performance for the correction (see Section 4.2.). These criteria are not provided by the OOINLCorrect Software [7] description.
- We used absorption units instead of percentage for the evaluation of the changes in the results (see Figure 10). The example data show the benefits of our approach.

## 5. Conclusions

As the use of compact charge-coupled device (CCD) spectrometers becomes more widespread and the applications become more demanding, the non-linearity correction method presented in this study will play an important role in improving the signal-to-noise ratio of these detectors. We described a simple experimental approach, without the need for special sophisticated components, for compensating for the error related to non-linearity. The data to derive the non-linearity correction function was collected by varying the integration time at a stable light emission. According to the experimental data, a one-degree change in temperature of the spectrometer resulted in a change in the ADC offset of about 2.4 counts. Therefore, the experiment was conducted at a constant temperature. Following statistical analysis, an intensity correction function was obtained that only depends on the intensity of the signal and is independent of integration time and wavelength. The whole method can optionally be performed without removing the spectrometer from an existing experimental setup.

We proposed a simple non-linearity correction method for CCD spectrometers to improve the signal-to-noise ratio of the readings. The error due to the non-linearity of a Hamamatsu C10082CA CCD spectrometer can be reduced from a systematic error of several hundred counts to a statistical error of about 40 counts at higher intensities with the correction function applied in this study and thus can increase the accuracy of the measurements significantly. This increase in accuracy is especially useful for applications that use extinction coefficients from the literature or rely on the absorption ratio of two or more components in a mixture. In those cases, a change in the intensity of the light source or a change in the integration time may yield different results. 

## Figures and Tables

**Table 1:** Characteristics of the spectrometer used, as provided by the manufacturer (Hamamatsu) [1,29].

Parameter	Hamamatsu C10082CA, S10420-1106-01 Series
Built-in sensor	Back-thinned CCD image sensor
Spectral range	200–800 nm
Number of pixels	2048
A/D conversion	16-bits
Integration time	10 to 10,000 ms
Operating temperature	+5 to +40 °C
Cooling	Non-cooled CCD
Blooming	Anti-blooming function applied
